# The development of a digital NGO mapping platform in Tanzania, as a tool to strengthen civil society engagement, in pursuit of universal health coverage

**DOI:** 10.11604/pamj.2023.46.115.42333

**Published:** 2023-12-22

**Authors:** Violet Wairimu Mathenge, Richard Sambaiga, Ally Bitebo, Modesta Komba, Abraham Mushashu, Mussa Sang'anya, Faki Shaweji, Grace Mbwilo, Vickness Mayao, Jerry Mlembwa, Jaliath Rangi, Chima Onuekwe, Neema Kileo, William Mwengee, Charles Sagoe-Moses

**Affiliations:** 1 World Health Organization, Tanzania Country Office, Dar es Salaam, Tanzania,; 2 The University of Dar es Salaam, Dar es Salaam, Tanzania,; 3 Ministry of Community Development, Gender, Women and Special Groups, Dodoma, Tanzania,; 4 World Health Organization, Inter-Country Support Team - East and Southern Africa, Harare, Zimbabwe

**Keywords:** Civil society, non-governmental organizations, digital mapping, universal health coverage, Tanzania

## Abstract

The role of civil society in economic development, improving livelihoods and in providing pathways towards achieving health for all has become increasingly evident. By mapping these organizations, the scope and scale as well as existing capacities, gaps, and opportunities are brought to light. This paper describes the implementation of a digital mapping platform for NGOs; an interactive site which collects, analyses, and visualizes data from a variety of sources about NGOs in Tanzania, through a series of interactive maps, graphs, and charts. We describe the approach and the technology used to develop the platform and its potential contribution towards improving health outcomes. A situation analysis and needs assessment exercise was conducted in February 2023. The developed system requirement specification document served as the guiding document in the design and development of new modules. Participatory techniques and agile iterative methodologies comprising regular stakeholder engagement were employed. A distributed revision control system was used to keep track of system revisions. The modules were deployed to the production server at the National Internet Data Center (NIDC) server room, followed by a system commissioning activity in October 2023. The NGO Information System, NGO Digital Mapping tool, NGO Analytic tool, NGO Search tool and NGO Opportunities module were designed, developed, and commissioned to support NGO operations in Tanzania. The platform was launched during the annual NGO Forum in Dodoma, Tanzania, on October 5, 2023. The modules are publicly accessible and are housed within the NGO Information System (NIS) platform. Investment in whole-of-society engagement to build health systems resilience for universal health coverage is crucial. Leveraging the unique positioning of NGOs draws us a step closer to the ambitious goal of achieving health for all. Through this one-stop web application system, information on the near real-time status, existing gaps, and opportunities for collaboration to serve communities is readily available for all stakeholders. Wide dissemination and enhancement of utilization of the platform across all sectors is now needed, for data to truly inform action.

## Introduction

In recent years, the role of civil society in economic development, improving livelihoods, and in providing pathways towards achieving health for all has become increasingly evident [1]. In the health sector, their involvement has been a catalyst for change in the global fight against HIV/AIDS, the Ebola response in West Africa, global Polio eradication efforts, and recently, in the roll-out of COVID-19 vaccines [2-4]. Collaboration with these organizations has become a priority for governments, donors, and partners owing to their comparative advantage in providing effective and targeted interventions that consider local contextual needs and realities [5-7]. While the usefulness of civil society organizations in the Global South is well understood, real-time information detailing their activities is often fragmented and not easily accessible in many countries [8]. This results in suboptimal coordination, collaboration, and utilization of resources that are earmarked to serve communities. Initiatives to map these organizations enhance understanding of the organizational sectors, their scope and scale as well as the existing capacities, gaps, and opportunities [9]. With technology, the possibility to digitize and analyze information in new ways and to utilize mapping as a tool for visibility, information sharing, strengthening collaboration, and enhancing transparency present endless opportunities for stakeholders and, notably, for communities [8].

The efforts towards digital health solutions in low and middle-income countries (LMICs) like Tanzania can hardly be ignored [10]. This is manifested by both the increased financial investment in digital technology in the health sector and its institutionalization through policies and strategies to govern both state and non-state actors. Available literature shows the potential of digital health in promoting efficient and responsive health systems [11-15]. It is further underlined that the digital health regime in Tanzania is well articulated in the current Tanzania Digital Health Strategy 2019-2024 and the Tanzania Digital Health Investment Road Map (2017-2023) [16,17]. These policy documents articulate the government´s desire to improve the application of digital health technologies to facilitate the delivery of high-quality health services to all citizens. To situate motivation for digital health interventions in Tanzania, it is important to highlight the broad ambition towards integration of Information and Communication Technology (ICT) in government affairs. Bwalya 2018 describes Tanzania as among the countries in Africa considered the leaders of e-Government implementation. Indeed, the country is one of the pioneers in institutionalization of e-government in the continent after having developed the 2013 Tanzania e-Government Strategy and formalizing many of the e-Government efforts [18].

Non-Governmental Organizations (NGO), (whose definition by Tanzania law includes Community-Based Organizations), form a significant proportion of Civil Society Organizations (CSO); a general concept used to cover all actors of civil society [19]. Despite efforts by the Ministry of Community Development, Gender, Women, and Special Groups (MCDGWSG) to strengthen coordination and information exchange, there have been challenges in accessing real-time information on the organizational landscape. While 10,155 NGOs are registered as of November 2023 -more than 30% of these falling within the health sector-there is a lack of clarity on their location, scope, area of work, experience, and impact on the population. Hence, collaboration with and engagement of these groups in reaching communities to provide and generate demand for life-saving interventions, such as immunization, has been suboptimal. To address these challenges, the MCDGWSG in partnership with the University of Dar es Salaam and with support from the Embassy of Switzerland and the World Health Organization, designed, developed, pretested, and activated a digital NGO mapping platform for all NGOs operating in Tanzania Mainland. This was an iterative process engaging various government ministries and sectors, as well as non-government stakeholders. Despite the growing research on digital health and e-government in general, little is known about what it takes to digitally map NGOs working in different sectors, including health in resource-constrained settings. In this paper, we draw evidence from Tanzania to showcase the power of participatory approaches involving both government actors and non-government stakeholders in effective processes of designing, developing, and deploying a user-friendly publicly accessible NGO information system readily available for all stakeholders. We discuss its utility in detailing and analyzing the NGO ecosystem, thereby facilitating effective coordination, and strengthening stakeholder engagement and collaboration in the health sector, where the role of civil society in reaching communities and generating demand for health services is paramount.

## Methods

**Situation analysis and needs assessment:** experts from the University of Dar es Salaam (UDSM) and the MCDGWSG conducted an operational review of the existing NGO Information System (NIS) in February 2023. This process included a review of system manuals and reports, and discussions on system functions and requirements with officers from the office of the registrar at the MCDGWSG. Based on the identified gaps and needs, a Software Requirement Specification (SRS) document was developed, with compliance with the e-Government Authority (e-GA) standards and guidelines for government information technology infrastructure and systems [20].

**Figure 1:**
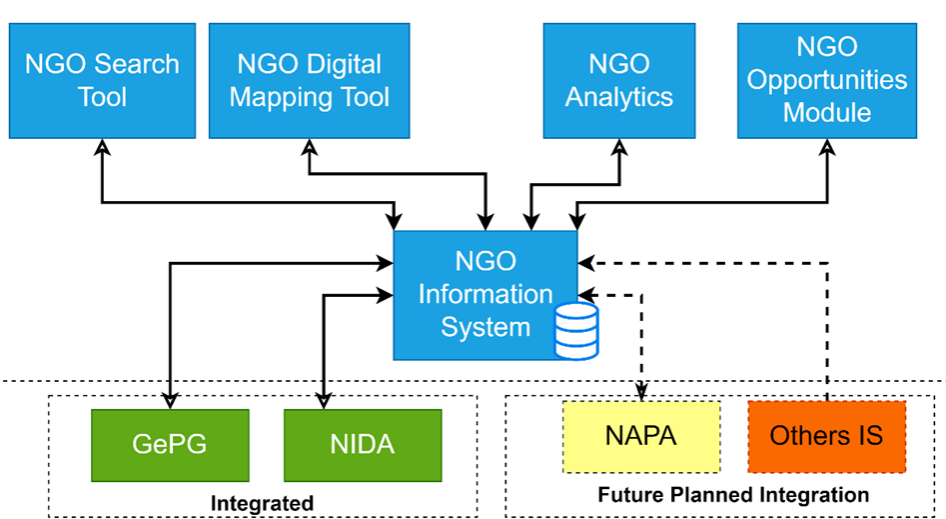
NGO information system (NIS) high level system architecture design

**Platform design:** building on the previous successful collaboration with the assistant registrars, the Office of the Registrar of NGOs partnered with the University of Dar es Salaam to design and develop the NGO Digital Mapping platform. The project drew experts from the Department of Sociology and Anthropology, and the Department of Computer Science and Engineering, who had prior experience in the development of dashboards for the Ministry. The SRS served as the guiding document for NIS system improvement and in the design and development of new modules housed within the system. During this phase, the physical and logical design of all NIS system modules and reports about user roles and privileges were designed and improved accordingly. Five modules, namely: the NGO information System module, NGO Search tool, NGO Mapping tool, NGO Analytic tool, and the NGO Opportunities module were designed and developed. Participatory techniques and agile iterative methodologies requiring regular stakeholder engagement were used. A distributed revision control system (DRCS) was used to keep track of system revisions and to deploy all developed and tested features to the testing server. The development of the NIS system modules was conducted in parallel with other analyses and design activities to ensure the timely availability of various project deliverables. Therefore, the back-end development of the NIS was developed by using Laravel framework; an open-source Hypertext Preprocessor (PHP) web development framework [21,22]. The Digital Mapping Tool module was developed by using Leaflet, which is an open-source JavaScript library used to build web mapping applications [23]. The NGO analytics tool was developed using custom-designed JavaScript code using the jQuery library [24]. The NIS system was integrated with the Government Electronic Payment Gateway (GePG) for online billing and payment and with the National Identification Authority (NIDA) system for verification of user National Identification Numbers (NIN) during NGO registration and change of particulars. The high-level system architecture is shown in Figure 1.

**Figure 2:**
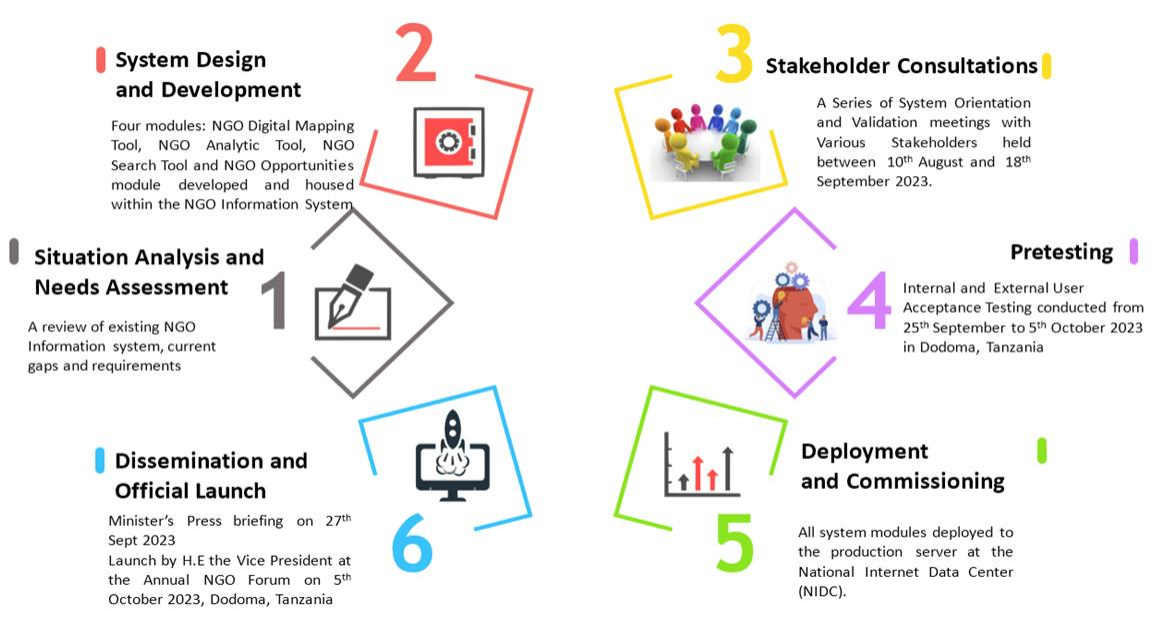
stages of development of the digital NGO mapping platform

**Stakeholder consultations:** we held a series of stakeholder consultative meetings with various stakeholders for information sharing, knowledge exchange, experience sharing, quality assurance, validation and to explore avenues for possible integration with other existing government platforms as follows: an initial multisectoral orientation meeting with NGO desk ministerial officers from 15 ministries in Tanzania mainland including the Ministry of Health and the President's Office, Regional Administration and Local Government (PO-RALG) was held in August 2023. This was followed by validation meetings with NaCoNGO, the NGO coordination board, and donors and development partners in Dar es Salaam (Figure 2). In September 2023, a hybrid meeting for experience sharing, knowledge exchange, and exploration of avenues of integration with existing government systems with various ministries, including Finance, Home Affairs, Communication and Information Technology, and agencies such as the PO- RALG, Financial Intelligence Unit (FIU), National Social Security Fund (NSSF), The Registration, Insolvency and Trusteeship Agency (RITA) and e-GA was held. Subsequently, a ministry quality assurance meeting chaired by the Deputy Permanent Secretary, MCDGWG to validate the NGO digital mapping platform was held on September 17, 2023. The platform was then presented to the Parliamentary Standing Committee on Social Welfare and Community Development for validation.

**System deployment:** all system modules: the improved NIS module, NGO Digital Mapping tool, NGO Analytic tool, NGO Search tool, and NGO opportunities module, were deployed to the production server at the National Internet Data Center (NIDC) server room with all features required to support the NGO management. This was followed by a system commissioning activity in October 2023.

**User acceptance testing:** user acceptance testing (UAT) was conducted over 5 days in September 2023 and completed at the Annual NGO Forum in Dodoma, Tanzania on October 4 - 5, 2023. A total of 103 test cases were recorded and discussed, to assess the system´s capacity to meet the development specifications.

**Dissemination and official launch:** a press briefing was held by the Minister on September 27, 2023, for wide dissemination of information on the platform. This was followed by a launch during the annual NGO Forum in Dodoma, Tanzania, on October 5, 2023.

## Results

**Figure 3:**
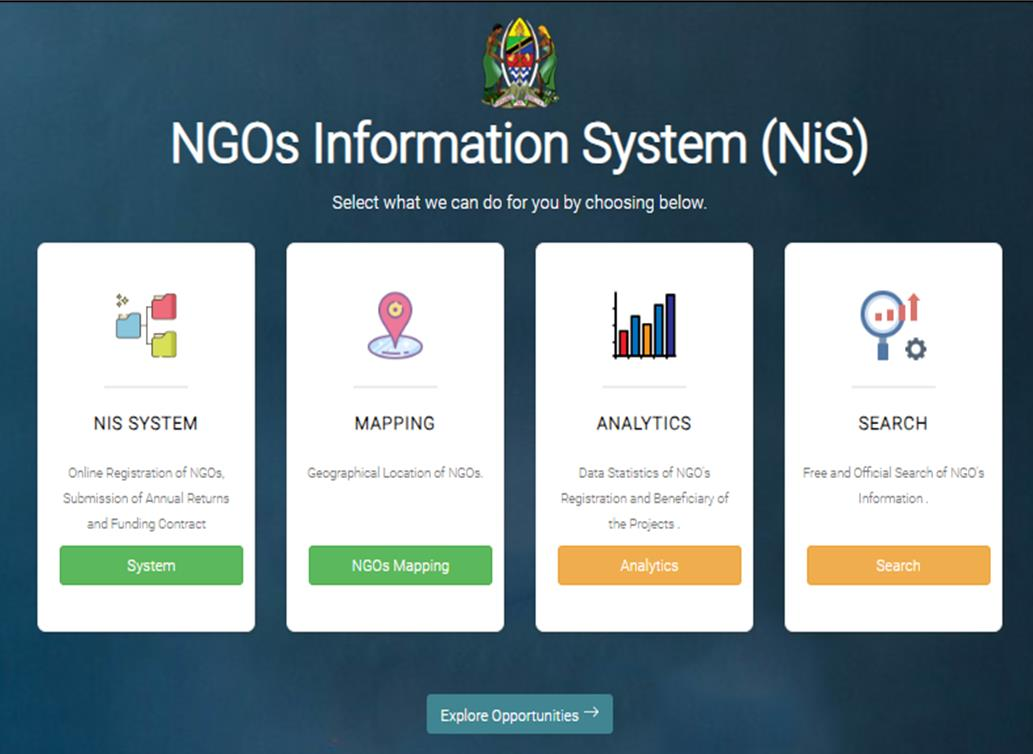
the NGO information system landing page

The NGO information system module, NGO Digital Mapping tool, NGO Analytic tool, NGO Search tool, and NGO Opportunities module were designed, developed, and deployed to support NGO operations in Tanzania. The modules are publicly accessible and are housed within the NGO Information System (NIS) platform (Figure 3).

**Figure 4:**
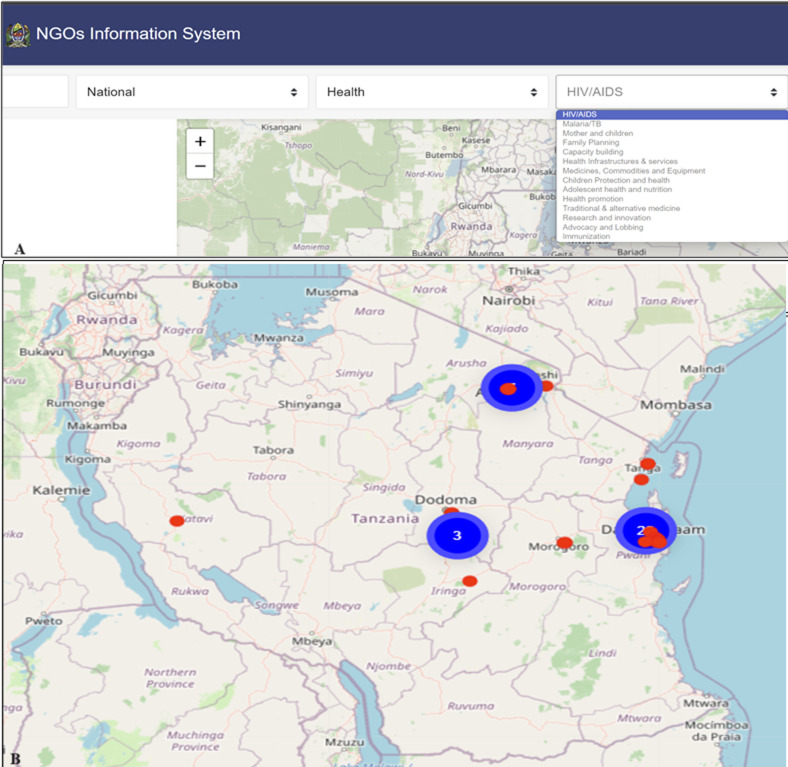
screen captures of the NGO mapping tool; A) health sectors filters, B) NGO mapping tool

**NGO digital mapping tool module:** the NGO digital mapping tool is linked to the data stored in the NIS module. The module plots NGO office's physical location based on geospatial data and maps areas of project implementation based on submitted annual and quarterly project reports. The exact NGO office location and aggregate numbers of NGOs in proximity are mapped using red and blue dots respectively. Additionally, the tool offers several search functionalities where users can filter data displayed on the map in real time by using the NGO name, operating level, region, district, sector, and sub-sector (Figure 4).

**Figure 5:**
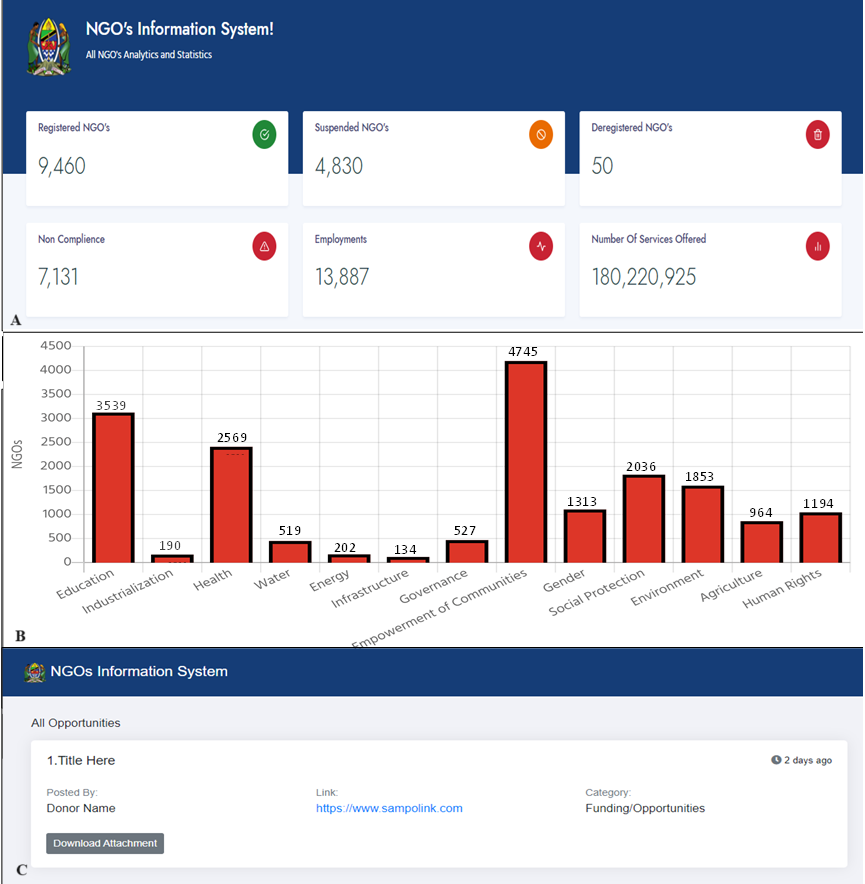
various screen captures taken from the NGO information system (NIS) dashboard web interface; A) NGO analytic tool dashboard, B) number of NGOs by sector, C) NGO opportunities tool

**NGO analytic tool module:** this module summarizes and displays key NGO information in the form of tables, charts, and graphs on a visual interactive dashboard. Analytical summaries based on key variables such as the number of registered NGOs, number of suspended and deregistered NGOs, employment created, services offered, and a list of non-compliant NGOs are displayed (Figure 5).

**Figure 6:**
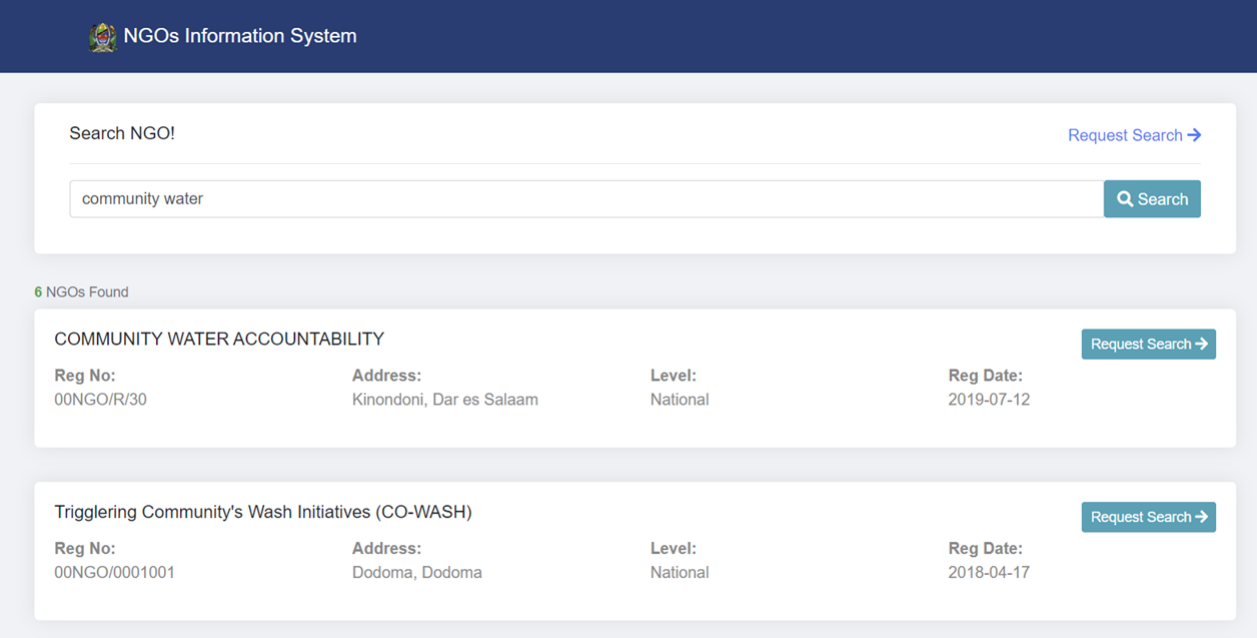
screen capture of the non-governmental organizations (NGO) search tool

**NGO search tool and opportunities module:** the NGO Search module manages and supports the search of NGO information stored in the NIS module. This includes the search for basic NGO information such as NGO names, registration numbers, registration dates, operational level, and address. Further, the module provides an option for users to request more information from the office of the registrar of NGOs through the request official search feature (Figure 6). The opportunities module displays employment opportunities and calls for proposals submitted by stakeholders to the Ministry.

## Discussion

A user-friendly publicly accessible platform detailing NGO location, sector, and subsector, and analyzing the NGO ecosystem by various parameters was successfully developed. This platform has multiple benefits: first, it will build the Ministry´s muscle in incoordination, monitoring, and evaluation of organizations´ capacities and performance in delivering various projects to communities. Second, it will provide key information to all stakeholders, thereby creating opportunities for partnership and strengthening the institutional capacities. Third, it will optimize the utilization of resources by reducing duplication of activities and presenting opportunities for cooperation among and within organizations with similar characteristics and priorities. These will contribute to the growth of the sector, in line with the national strategy for NGO sustainability that focuses on the execution of need-based, and demand-driven programs, supported by excellent leadership, transparency, accountability, and commitment to sustainability and meeting expressed needs of beneficiaries [25,26]. We found that the iterative process, involving multiple stakeholders from the design phase, was key in fostering ownership, creating awareness, and ensuring that the developed system was useful and relevant. Indeed, entities such as NaCoNGO, -established for coordination and networking- and the NGO coordination board were instrumental in providing essential inputs during system development [27]. In addition, the involvement of other government sectors with similar functional systems enhanced experience sharing and exchange of lessons learned. Integration with GePG and NIDA systems improved process efficiency by creating avenues for seamless data exchange.

The platform was timely for the health sector, given the lessons from the COVID-19 pandemic on the critical role communities and civil society can play in responding to public health emergencies and in maintaining essential health services [28]. While CSOs across African countries were widely engaged in the response, one of the main drawbacks observed was the inadequacy of requisite skills and expertise in the implementation of interventions [29]. From this experience, there is heightened emphasis on investment in whole-of-society engagement to build health systems resilience for universal health coverage [30]. Using this platform, plans for systematic identification, profiling, and selection of organizations for capacity building and engagement in key interventions such as immunization are underway. Nevertheless, we had some limitations; first, inadequate knowledge of the registration processes has resulted in under registration of organizations that otherwise qualify to be identified as NGOs as per the NGOs Act, Cap 56. This, coupled with the underutilization of the pre-existing system for registration and reporting, interfered with the completeness of the data. Second, while the system has the capacity to display and analyze the Community-Based Organizations in the country, uploading those records onto the system was delayed. Despite these limitations, the orientation of assistant registrars across all regions on how to upload information onto the system is ongoing.

## Conclusion

In conclusion, investment in whole-of-society engagement to build health systems resilience for universal health coverage is crucial. Leveraging the unique positioning of NGOs at the heart of communities draws us a step closer to the ambitious goal of achieving health for all. Through this one-stop web application system, information on the near real-time status, existing gaps, and opportunities for collaboration to serve communities is readily available for all stakeholders. Wide dissemination and enhancement of utilization of the platform across all sectors is now needed, for data to truly inform action.
